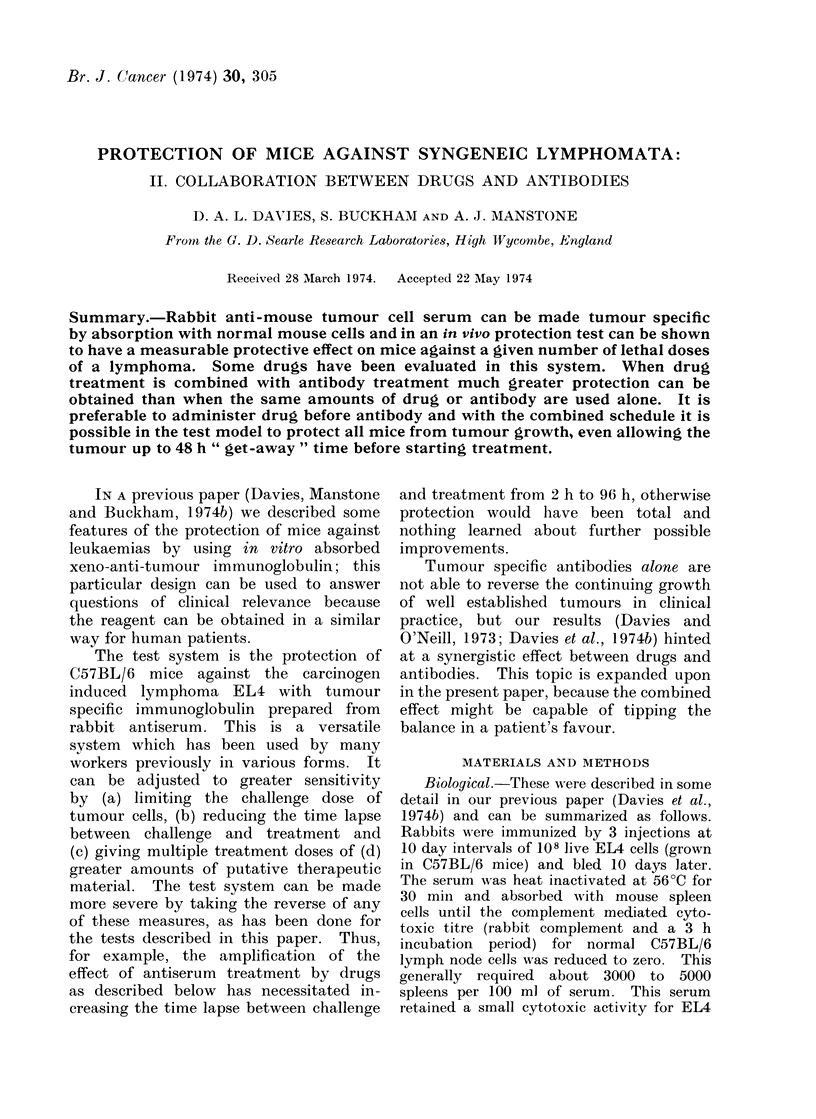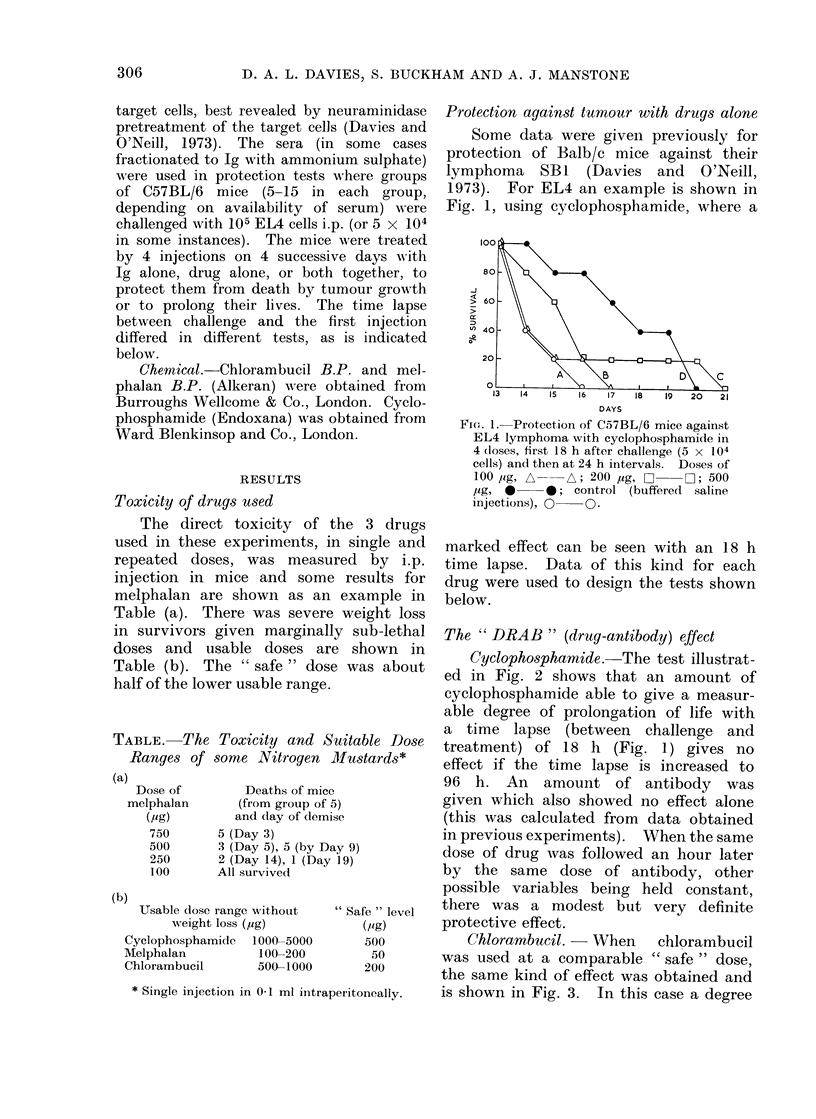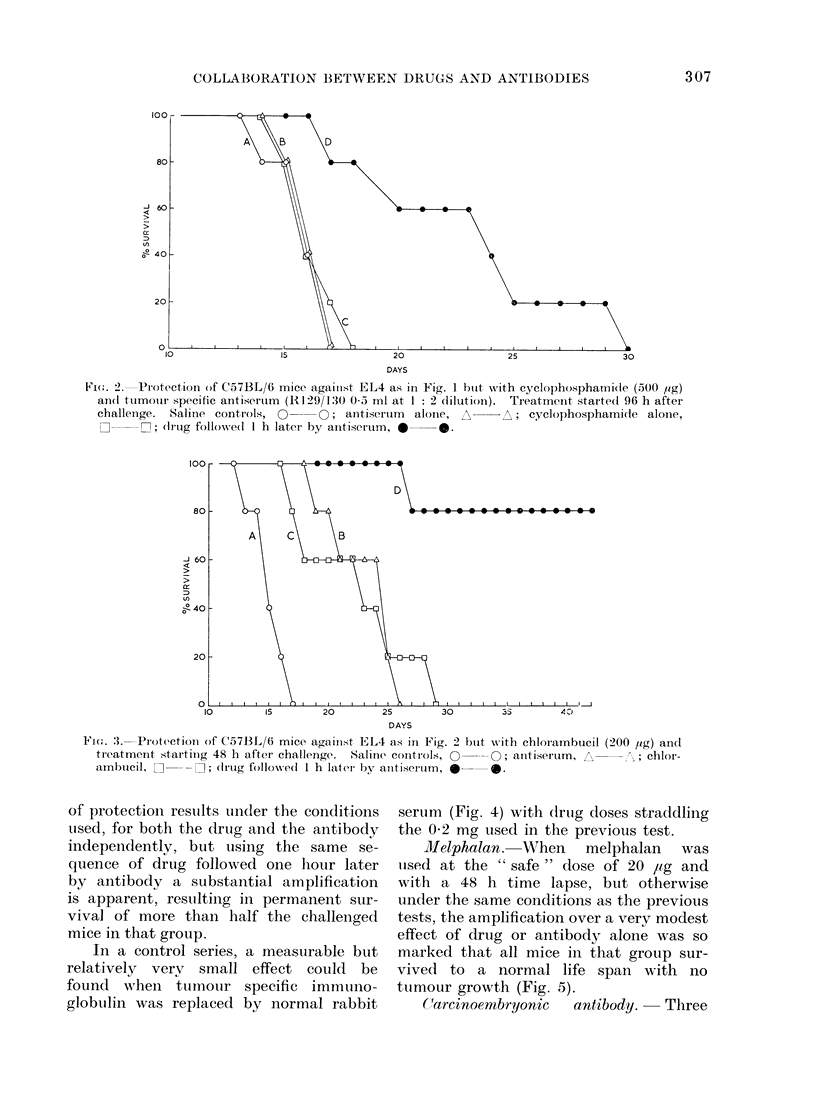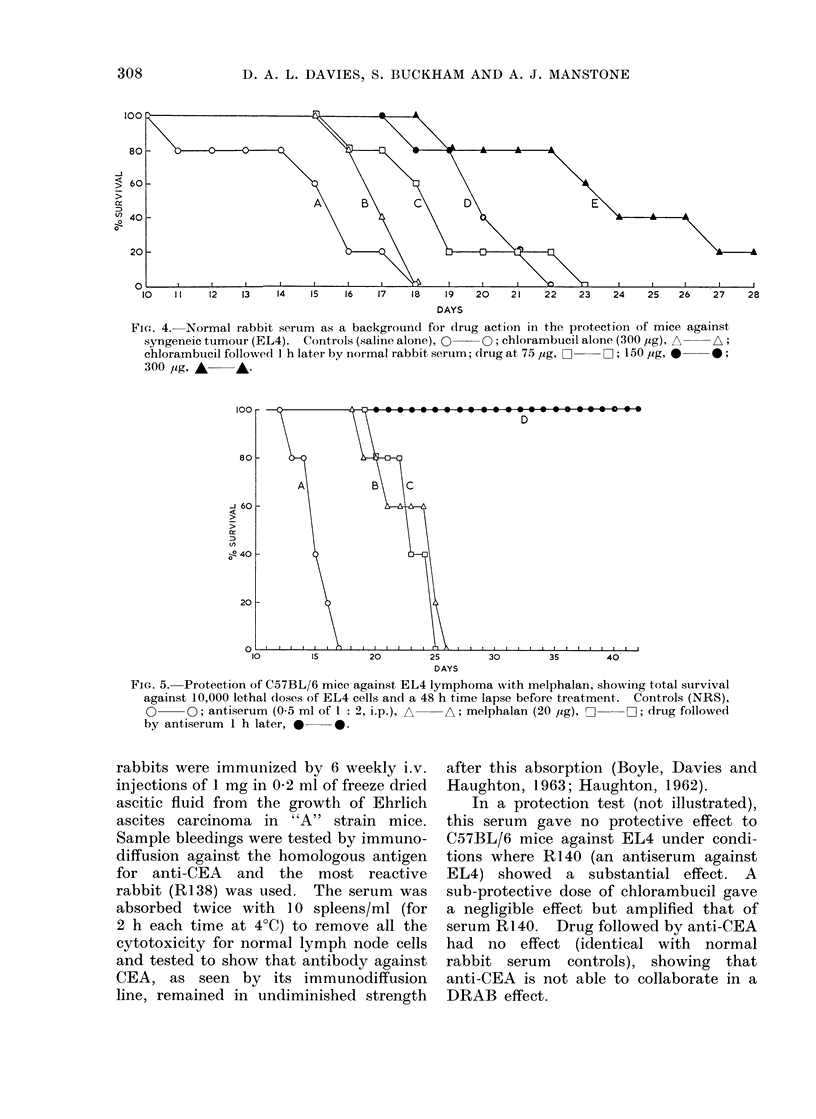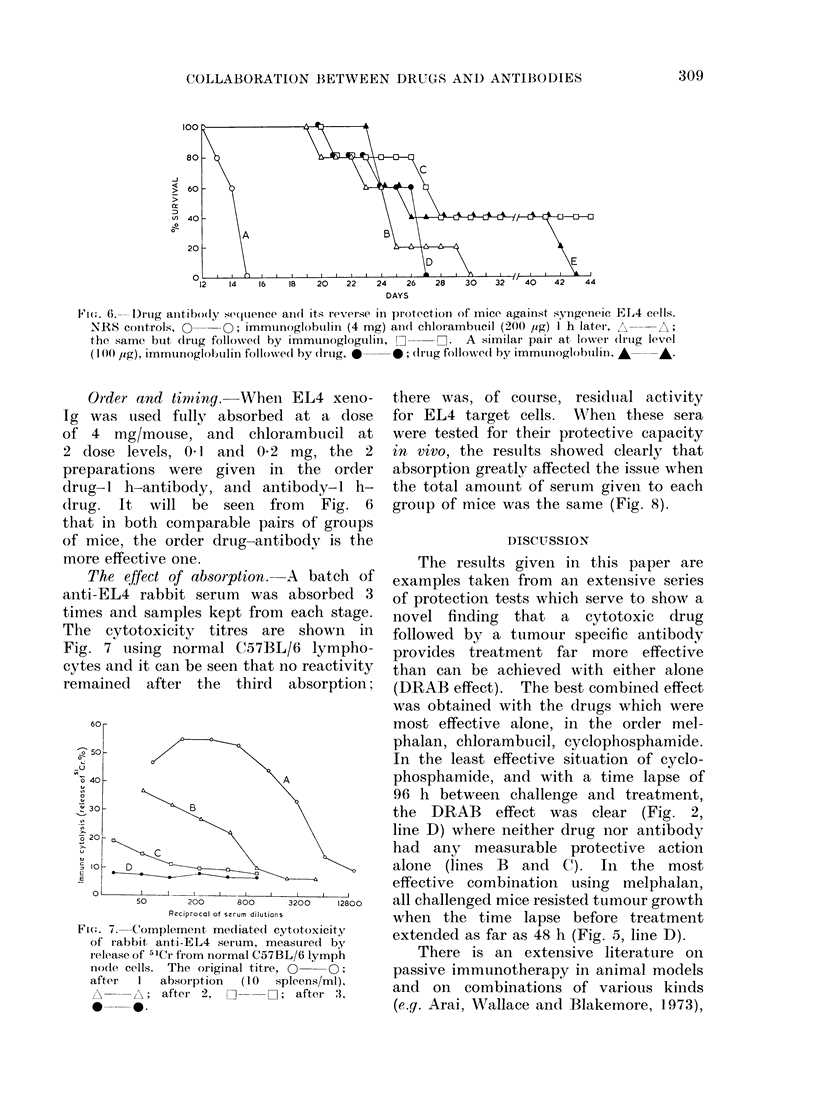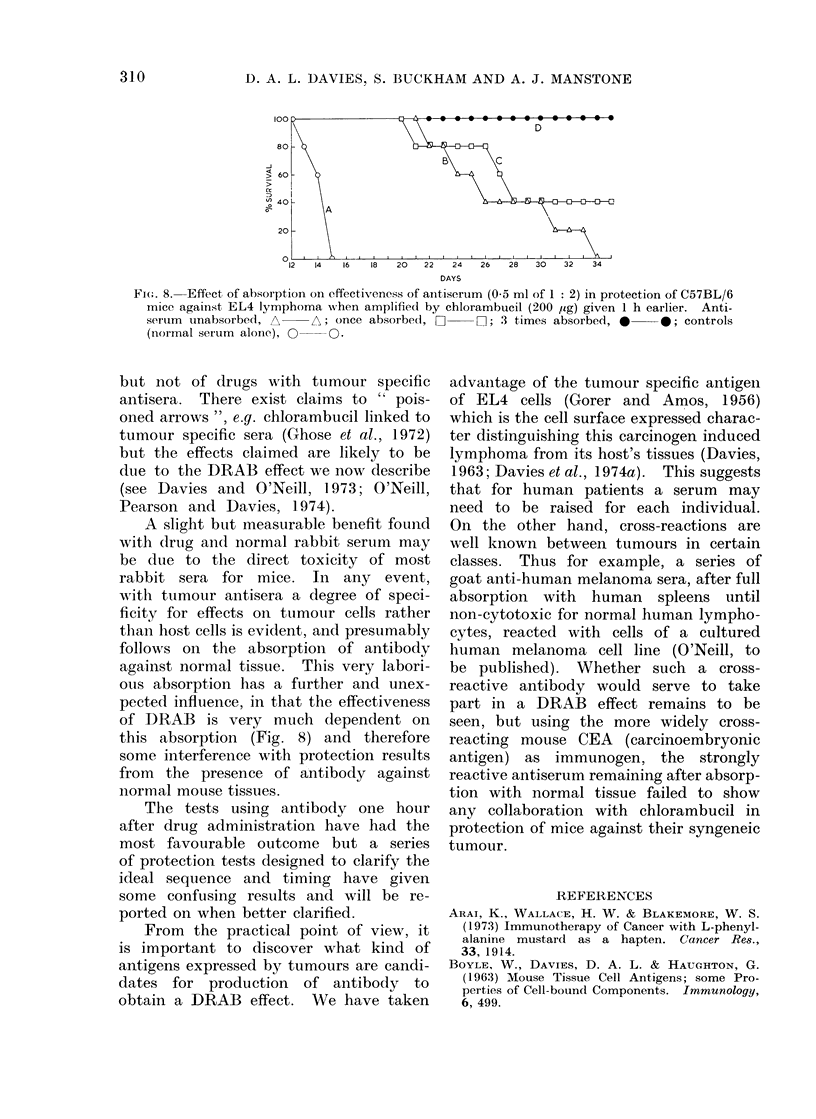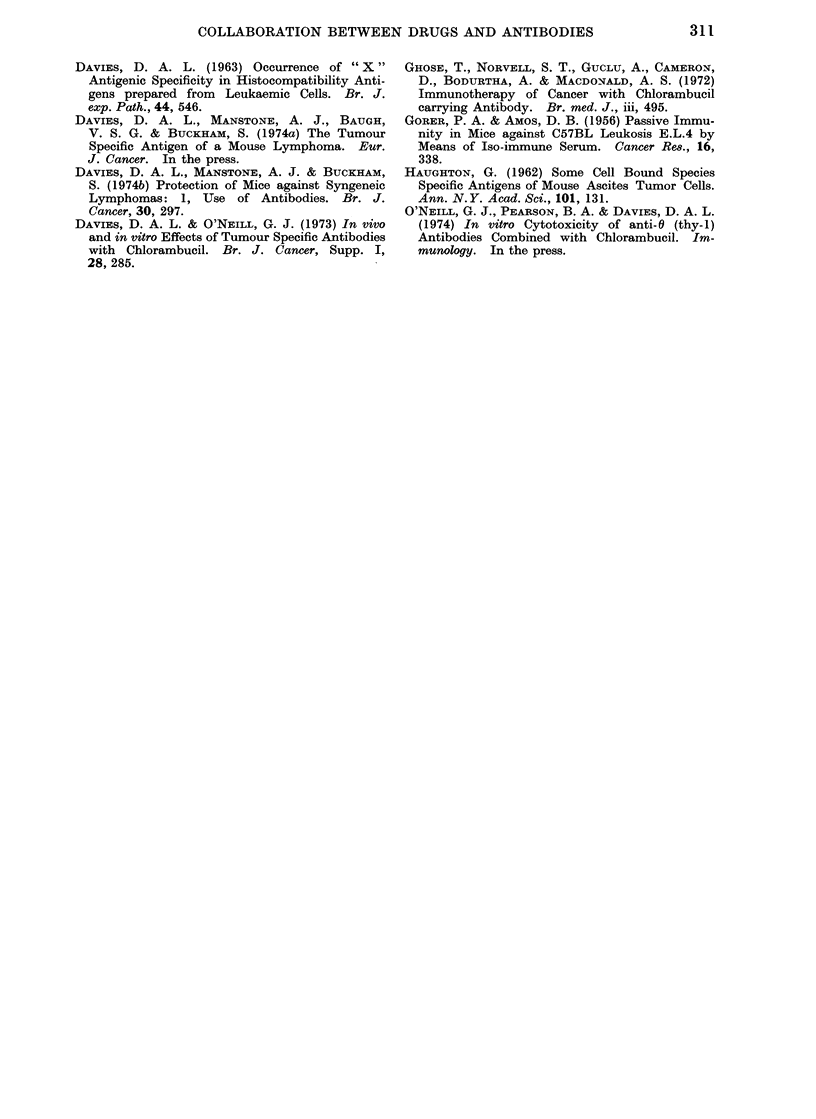# Protection of Mice Against Syngeneic Lymphomata

**DOI:** 10.1038/bjc.1974.197

**Published:** 1974-10

**Authors:** D. A. L. Davies, S. Buckham, A. J. Manstone

## Abstract

Rabbit anti-mouse tumour cell serum can be made tumour specific by absorption with normal mouse cells and in an *in vivo* protection test can be shown to have a measurable protective effect on mice against a given number of lethal doses of a lymphoma. Some drugs have been evaluated in this system. When drug treatment is combined with antibody treatment much greater protection can be obtained than when the same amounts of drug or antibody are used alone. It is preferable to administer drug before antibody and with the combined schedule it is possible in the test model to protect all mice from tumour growth, even allowing the tumour up to 48 h “get-away” time before starting treatment.


*Br. J. Cancer* (1974) **30**, 305

### PROTECTION OF MICE AGAINST SYNGENEIC LYMPHOMATA:
### II. COLLABORATION BETWEEN DRUGS AND ANTIBODIES

D. A. L. DAVIES, S. BUCKHAM AND A. J. MANSTONE

*From the G. D. Searle Research Laboratories, High Wycombe, England*

Received 28 March 1974. Accepted 22 May 1974

**Summary.—Rabbit anti-mouse tumour cell serum can be made tumour specific by absorption with normal mouse cells and in an *in vivo* protection test can be shown to have a measurable protective effect on mice against a given number of lethal doses of a lymphoma. Some drugs have been evaluated in this system. When drug treatment is combined with antibody treatment much greater protection can be obtained than when the same amounts of drug or antibody are used alone. It is preferable to administer drug before antibody and with the combined schedule it is possible in the test model to protect all mice from tumour growth, even allowing the tumour up to 48 h " get-away " time before starting treatment.**

IN A previous paper (Davies, Manstone and Buckham, 1974*b*) we described some features of the protection of mice against leukaemias by using *in vitro* absorbed xeno-anti-tumour immunoglobulin; this particular design can be used to answer questions of clinical relevance because the reagent can be obtained in a similar way for human patients.

The test system is the protection of C57BL/6 mice against the carcinogen induced lymphoma EL4 with tumour specific immunoglobulin prepared from rabbit antiserum. This is a versatile system which has been used by many workers previously in various forms. It can be adjusted to greater sensitivity by (a) limiting the challenge dose of tumour cells, (b) reducing the time lapse between challenge and treatment and (c) giving multiple treatment doses of (d) greater amounts of putative therapeutic material. The test system can be made more severe by taking the reverse of any of these measures, as has been done for the tests described in this paper. Thus, for example, the amplification of the effect of antiserum treatment by drugs as described below has necessitated increasing the time lapse between challenge and treatment from 2 h to 96 h, otherwise protection would have been total and nothing learned about further possible improvements.

Tumour specific antibodies *alone* are not able to reverse the continuing growth of well established tumours in clinical practice, but our results (Davies and O'Neill, 1973; Davies *et al.*, 1974*b*) hinted at a synergistic effect between drugs and antibodies. This topic is expanded upon in the present paper, because the combined effect might be capable of tipping the balance in a patient's favour.

### MATERIALS AND METHODS

*Biological.*—These were described in some detail in our previous paper (Davies *et al.*, 1974*b*) and can be summarized as follows. Rabbits were immunized by 3 injections at 10 day intervals of $10^8$ live EL4 cells (grown in C57BL/6 mice) and bled 10 days later. The serum was heat inactivated at 56°C for 30 min and absorbed with mouse spleen cells until the complement mediated cytotoxic titre (rabbit complement and a 3 h incubation period) for normal C57BL/6 lymph node cells was reduced to zero. This generally required about 3000 to 5000 spleens per 100 ml of serum. This serum retained a small cytotoxic activity for EL4

target cells, best revealed by neuraminidase pretreatment of the target cells (Davies and O'Neill, 1973). The sera (in some cases fractionated to Ig with ammonium sulphate) were used in protection tests where groups of C57BL/6 mice (5–15 in each group, depending on availability of serum) were challenged with $10^5$ EL4 cells i.p. (or $5 \times 10^4$ in some instances). The mice were treated by 4 injections on 4 successive days with Ig alone, drug alone, or both together, to protect them from death by tumour growth or to prolong their lives. The time lapse between challenge and the first injection differed in different tests, as is indicated below.

*Chemical.*—Chlorambucil *B.P.* and melphalan *B.P.* (Alkeran) were obtained from Burroughs Wellcome & Co., London. Cyclophosphamide (Endoxana) was obtained from Ward Blenkinsop and Co., London.

### RESULTS

### *Toxicity of drugs used*

The direct toxicity of the 3 drugs used in these experiments, in single and repeated doses, was measured by i.p. injection in mice and some results for melphalan are shown as an example in Table (a). There was severe weight loss in survivors given marginally sub-lethal doses and usable doses are shown in Table (b). The " safe " dose was about half of the lower usable range.

TABLE.—*The Toxicity and Suitable Dose Ranges of some Nitrogen Mustards**

(a)

| Dose of melphalan (μg) | Deaths of mice (from group of 5) and day of demise |
|---|---|
| 750 | 5 (Day 3) |
| 500 | 3 (Day 5), 5 (by Day 9) |
| 250 | 2 (Day 14), 1 (Day 19) |
| 100 | All survived |

(b)

| | Usable dose range without weight loss (μg) | " Safe " level (μg) |
|---|---|---|
| Cyclophosphamide | 1000–5000 | 500 |
| Melphalan | 100–200 | 50 |
| Chlorambucil | 500–1000 | 200 |

* Single injection in 0·1 ml intraperitoneally.

### *Protection against tumour with drugs alone*

Some data were given previously for protection of Balb/c mice against their lymphoma SB1 (Davies and O'Neill, 1973). For EL4 an example is shown in Fig. 1, using cyclophosphamide, where a

FIG. 1.—Protection of C57BL/6 mice against EL4 lymphoma with cyclophosphamide in 4 doses, first 18 h after challenge ($5 \times 10^4$ cells) and then at 24 h intervals. Doses of 100 μg, △——△; 200 μg, □——□; 500 μg, ●——●; control (buffered saline injections), ○——○.

marked effect can be seen with an 18 h time lapse. Data of this kind for each drug were used to design the tests shown below.

### *The " DRAB " (drug-antibody) effect*

*Cyclophosphamide.*—The test illustrated in Fig. 2 shows that an amount of cyclophosphamide able to give a measurable degree of prolongation of life with a time lapse (between challenge and treatment) of 18 h (Fig. 1) gives no effect if the time lapse is increased to 96 h. An amount of antibody was given which also showed no effect alone (this was calculated from data obtained in previous experiments). When the same dose of drug was followed an hour later by the same dose of antibody, other possible variables being held constant, there was a modest but very definite protective effect.

*Chlorambucil.* — When chlorambucil was used at a comparable " safe " dose, the same kind of effect was obtained and is shown in Fig. 3. In this case a degree

Fig. 2.—Protection of C57BL/6 mice against EL4 as in Fig. 1 but with cyclophosphamide (500 $\mu$g) and tumour specific antiserum (R129/130 0·5 ml at 1 : 2 dilution). Treatment started 96 h after challenge. Saline controls, ○——○; antiserum alone, △——△; cyclophosphamide alone, □——□; drug followed 1 h later by antiserum, ●——●.

Fig. 3.—Protection of C57BL/6 mice against EL4 as in Fig. 2 but with chlorambucil (200 $\mu$g) and treatment starting 48 h after challenge. Saline controls, ○——○; antiserum, △——△; chlorambucil, □——□; drug followed 1 h later by antiserum, ●——●.

of protection results under the conditions used, for both the drug and the antibody independently, but using the same sequence of drug followed one hour later by antibody a substantial amplification is apparent, resulting in permanent survival of more than half the challenged mice in that group.

In a control series, a measurable but relatively very small effect could be found when tumour specific immunoglobulin was replaced by normal rabbit serum (Fig. 4) with drug doses straddling the 0·2 mg used in the previous test.

*Melphalan*.—When melphalan was used at the " safe " dose of 20 $\mu$g and with a 48 h time lapse, but otherwise under the same conditions as the previous tests, the amplification over a very modest effect of drug or antibody alone was so marked that all mice in that group survived to a normal life span with no tumour growth (Fig. 5).

*Carcinoembryonic antibody*. — Three

FIG. 4.—Normal rabbit serum as a background for drug action in the protection of mice against syngeneic tumour (EL4). Controls (saline alone), ○——○; chlorambucil alone (300 μg), △——△; chlorambucil followed 1 h later by normal rabbit serum; drug at 75 μg, □——□; 150 μg, ●——●; 300 μg, ▲——▲.

FIG. 5.—Protection of C57BL/6 mice against EL4 lymphoma with melphalan, showing total survival against 10,000 lethal doses of EL4 cells and a 48 h time lapse before treatment. Controls (NRS), ○——○; antiserum (0·5 ml of 1 : 2, i.p.), △——△; melphalan (20 μg), □——□; drug followed by antiserum 1 h later, ●——●.

rabbits were immunized by 6 weekly i.v. injections of 1 mg in 0·2 ml of freeze dried ascitic fluid from the growth of Ehrlich ascites carcinoma in "A" strain mice. Sample bleedings were tested by immunodiffusion against the homologous antigen for anti-CEA and the most reactive rabbit (R138) was used. The serum was absorbed twice with 10 spleens/ml (for 2 h each time at 4°C) to remove all the cytotoxicity for normal lymph node cells and tested to show that antibody against CEA, as seen by its immunodiffusion line, remained in undiminished strength after this absorption (Boyle, Davies and Haughton, 1963; Haughton, 1962).

In a protection test (not illustrated), this serum gave no protective effect to C57BL/6 mice against EL4 under conditions where R140 (an antiserum against EL4) showed a substantial effect. A sub-protective dose of chlorambucil gave a negligible effect but amplified that of serum R140. Drug followed by anti-CEA had no effect (identical with normal rabbit serum controls), showing that anti-CEA is not able to collaborate in a DRAB effect.

FIG. 6.—Drug antibody sequence and its reverse in protection of mice against syngeneic EL4 cells. NRS controls, ○——○; immunoglobulin (4 mg) and chlorambucil (200 μg) 1 h later, △——△; the same but drug followed by immunoglogulin, □——□. A similar pair at lower drug level (100 μg), immunoglobulin followed by drug, ●——●; drug followed by immunoglobulin, ▲——▲.

*Order and timing.*—When EL4 xeno-Ig was used fully absorbed at a dose of 4 mg/mouse, and chlorambucil at 2 dose levels, 0·1 and 0·2 mg, the 2 preparations were given in the order drug–1 h–antibody, and antibody–1 h–drug. It will be seen from Fig. 6 that in both comparable pairs of groups of mice, the order drug–antibody is the more effective one.

*The effect of absorption.*—A batch of anti-EL4 rabbit serum was absorbed 3 times and samples kept from each stage. The cytotoxicity titres are shown in Fig. 7 using normal C57BL/6 lymphocytes and it can be seen that no reactivity remained after the third absorption; there was, of course, residual activity for EL4 target cells. When these sera were tested for their protective capacity *in vivo*, the results showed clearly that absorption greatly affected the issue when the total amount of serum given to each group of mice was the same (Fig. 8).

FIG. 7.—Complement mediated cytotoxicity of rabbit anti-EL4 serum, measured by release of $^{51}$Cr from normal C57BL/6 lymph node cells. The original titre, ○——○; after 1 absorption (10 spleens/ml), △——△; after 2, □——□; after 3, ●——●.

### DISCUSSION

The results given in this paper are examples taken from an extensive series of protection tests which serve to show a novel finding that a cytotoxic drug followed by a tumour specific antibody provides treatment far more effective than can be achieved with either alone (DRAB effect). The best combined effect was obtained with the drugs which were most effective alone, in the order melphalan, chlorambucil, cyclophosphamide. In the least effective situation of cyclophosphamide, and with a time lapse of 96 h between challenge and treatment, the DRAB effect was clear (Fig. 2, line D) where neither drug nor antibody had any measurable protective action alone (lines B and C). In the most effective combination using melphalan, all challenged mice resisted tumour growth when the time lapse before treatment extended as far as 48 h (Fig. 5, line D).

There is an extensive literature on passive immunotherapy in animal models and on combinations of various kinds (*e.g.* Arai, Wallace and Blakemore, 1973),

FIG. 8.—Effect of absorption on effectiveness of antiserum (0·5 ml of 1 : 2) in protection of C57BL/6 mice against EL4 lymphoma when amplified by chlorambucil (200 $\mu$g) given 1 h earlier. Antiserum unabsorbed, △——△; once absorbed, □——□; 3 times absorbed, ●——●; controls (normal serum alone), ○——○.

but not of drugs with tumour specific antisera. There exist claims to " poisoned arrows ", *e.g.* chlorambucil linked to tumour specific sera (Ghose *et al.*, 1972) but the effects claimed are likely to be due to the DRAB effect we now describe (see Davies and O'Neill, 1973; O'Neill, Pearson and Davies, 1974).

A slight but measurable benefit found with drug and normal rabbit serum may be due to the direct toxicity of most rabbit sera for mice. In any event, with tumour antisera a degree of specificity for effects on tumour cells rather than host cells is evident, and presumably follows on the absorption of antibody against normal tissue. This very laborious absorption has a further and unexpected influence, in that the effectiveness of DRAB is very much dependent on this absorption (Fig. 8) and therefore some interference with protection results from the presence of antibody against normal mouse tissues.

The tests using antibody one hour after drug administration have had the most favourable outcome but a series of protection tests designed to clarify the ideal sequence and timing have given some confusing results and will be reported on when better clarified.

From the practical point of view, it is important to discover what kind of antigens expressed by tumours are candidates for production of antibody to obtain a DRAB effect. We have taken advantage of the tumour specific antigen of EL4 cells (Gorer and Amos, 1956) which is the cell surface expressed character distinguishing this carcinogen induced lymphoma from its host's tissues (Davies, 1963; Davies *et al.*, 1974*a*). This suggests that for human patients a serum may need to be raised for each individual. On the other hand, cross-reactions are well known between tumours in certain classes. Thus for example, a series of goat anti-human melanoma sera, after full absorption with human spleens until non-cytotoxic for normal human lymphocytes, reacted with cells of a cultured human melanoma cell line (O'Neill, to be published). Whether such a cross-reactive antibody would serve to take part in a DRAB effect remains to be seen, but using the more widely cross-reacting mouse CEA (carcinoembryonic antigen) as immunogen, the strongly reactive antiserum remaining after absorption with normal tissue failed to show any collaboration with chlorambucil in protection of mice against their syngeneic tumour.

### REFERENCES

ARAI, K., WALLACE, H. W. & BLAKEMORE, W. S. (1973) Immunotherapy of Cancer with L-phenylalanine mustard as a hapten. *Cancer Res.*, **33**, 1914.

BOYLE, W., DAVIES, D. A. L. & HAUGHTON, G. (1963) Mouse Tissue Cell Antigens; some Properties of Cell-bound Components. *Immunology*, **6**, 499.

Davies, D. A. L. (1963) Occurrence of "X" Antigenic Specificity in Histocompatibility Antigens prepared from Leukaemic Cells. *Br. J. exp. Path.*, **44**, 546.

Davies, D. A. L., Manstone, A. J., Baugh, V. S. G. & Buckham, S. (1974*a*) The Tumour Specific Antigen of a Mouse Lymphoma. *Eur. J. Cancer*. In the press.

Davies, D. A. L., Manstone, A. J. & Buckham, S. (1974*b*) Protection of Mice against Syngeneic Lymphomas: 1, Use of Antibodies. *Br. J. Cancer*, **30**, 297.

Davies, D. A. L. & O'Neill, G. J. (1973) *In vivo* and *in vitro* Effects of Tumour Specific Antibodies with Chlorambucil. *Br. J. Cancer*, Supp. I, **28**, 285.

Ghose, T., Norvell, S. T., Guclu, A., Cameron, D., Bodurtha, A. & Macdonald, A. S. (1972) Immunotherapy of Cancer with Chlorambucil carrying Antibody. *Br. med. J.*, iii, 495.

Gorer, P. A. & Amos, D. B. (1956) Passive Immunity in Mice against C57BL Leukosis E.L.4 by Means of Iso-immune Serum. *Cancer Res.*, **16**, 338.

Haughton, G. (1962) Some Cell Bound Species Specific Antigens of Mouse Ascites Tumor Cells. *Ann. N.Y. Acad. Sci.*, **101**, 131.

O'Neill, G. J., Pearson, B. A. & Davies, D. A. L. (1974) *In vitro* Cytotoxicity of anti-$\theta$ (thy-1) Antibodies Combined with Chlorambucil. *Immunology*. In the press.